# Appendiceal ascariasis in children

**DOI:** 10.4103/0256-4947.59380

**Published:** 2010

**Authors:** Imtiaz Wani, Muddasir Maqbool, Abid Amin, Firdous Shah, Arshad Keema, Jang Singh, Maki Kitagawa, Mir Nazir

**Affiliations:** a From the Department of Surgery, SMHS Hospital, Srinagar, India; b From the GB Pant Hospital, Srinagar, India; c From the Department of Pathology, SMHS Hospital, Srinagar, India; d From the Department of Surgery, Shakaihoken Kobe Central Hospital, Kobe, Japan

## Abstract

**BACKGROUND:**

The propensity of *Ascaris lumbricoides* to wander leads to varied surgical complications in the abdomen. Wandering *A lumbricoides* may sometimes reach the vermiform appendix and its presence there may remain silent or incite pathology. Our aim was to study ascariadial appendicitis.

**METHODS:**

Over a period of 3 years, we identified children who were found to have appendiceal ascariasis during surgery for different intestinal complications due to ascariasis. We studied the relationship between ascariasis and its lodgement inside the vermiform appendix in these patients. No preoperative diagnosis was made in this series.

**RESULTS:**

We found 11 patients with appendiceal ascariasis. It was incidentally found that 8/11 (72.7%) patients had worms inside their vermiform appendix but not appendicitis, whereas the remaining three patients (27.2%) were found to have *Ascaris*-associated appendicitis. The characteristic finding in *Ascaris*-infested vermiform appendix was that the worm is positioned with its head at the base and its tail at the tip of the appendix.

**CONCLUSION:**

Migration of *A lumbrocoides* inside the vermiform appendix is an incidental finding and tends to pursue a silent course in most patients. Only rarely does the presence of *Ascaris* inside the vermiform appendix cause appendicitis.

*Ascaris lumbricoides* is rarely seen in the vermiform appendix although they are seen in the intestines of individuals in tropical countries. *Ascaris*-associated appendicitis is a form of wandering ascariasis and is usually the sequelae of a high intestinal worm load.^1^,^2^ *Ascaris* can be found in the normal appendix but may also be associated with appendicitis. We studied the clinical and pathological sequelae of the migration of *Ascaris* to the appendix.

## METHODS

Between May 2005 and May 2008, we identified children who were found to have appendiceal ascariasis during surgery for different intestinal complications due to ascariasis at SMHS Hospital. Age, sex, clinical features, operative diagnosis, and pathological findings confirmed by histological examination were recorded for patients whose vermiform appendix showed the presence of *Ascaris*.

## RESULTS

During a three-year period, a total of 11 patients with appendiceal ascariasis were encountered. Ten (91%) of these 11 patients were male and one was female (9%) (Table 1). Worms were found in the appendix during the course of surgery for ascaridial intestinal obstruction. Intestinal obstruction by worms was observed in seven patients (63.6%), adhesion obstruction (not related to *Ascaris*) in one patient (9%), appendicitis in two patients (18%), and peritonitis in one patient (9%).

**Table 1:** Patient characteristics and pathology of appendix.

Patient number	Age (years)	Sex	Symptoms	Preoperative diagnosis	Number of worms in the appendix	Treatment	Appendicitis
1	4	F	Abdominal pain, fever, vomiting, worms in the stool	Peritonitis	(5 worms in the peritoneal cavity)	Appendectomy	Yes
2	4	M	Abdominal pain, fever, vomiting of worms, constipation	Intestinal obstruction by worms	1	Enterotomy, appendectomy	No
3	4	M	Abdominal pain, vomiting of worms, worms in the stool	Intestinal obstruction by worms	1	Kneading, appendectomy	No
4	5	M	Abdominal pain, constipation	Intestinal obstruction by worms	1	Kneading, appendectomy	No
5	5	M	Abdominal pain, vomiting of worms, worms in the stool, diarrhea	Intestinal obstruction by worms	3	Kneading, appendectomy	No
6	6	M	Abdominal pain, fever, vomiting of worms, constipation	Intestinal obstruction by worms	1	Enterotomy, appendectomy	No
7	7	M	Abdominal pain, constipation	Intestinal obstruction by worms	1	Kneading, appendectomy	No
8	8	M	Right lower abdominal pain, fever, diarrhea	Appendicitis	1	Appendectomy	Yes
9	8	M	Abdominal pain, vomiting, constipation	Intestinal obstruction by worms	1	Kneading, appendectomy	No
10	11	M	Right lower abdominal pain, fever, worms in the stool	Appendicitis	1	Appendectomy	Yes
11	12	M	Abdominal pain, vomiting, constipation	Intestinal obstruction by adhesion	1	Adhesionolysis, appendectomy	No

**Figure 1:**
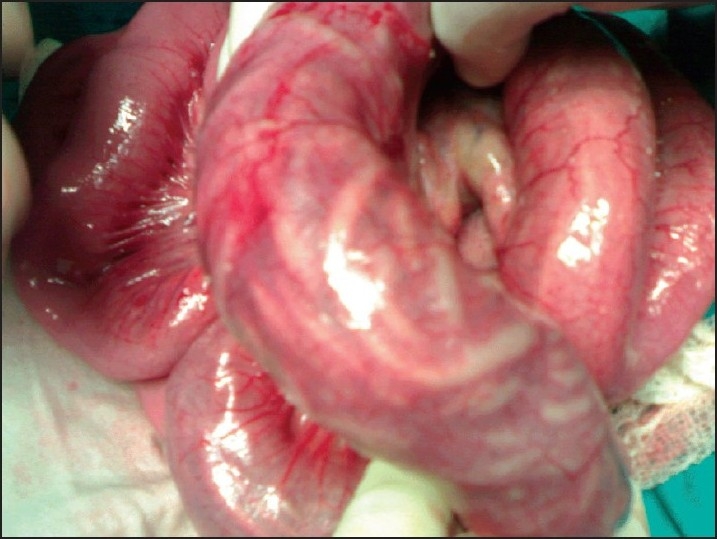
Long impacted worm bolus with transerosal visbility in a child who had incidental finding of worm inside appendix.

**Figure 2:**
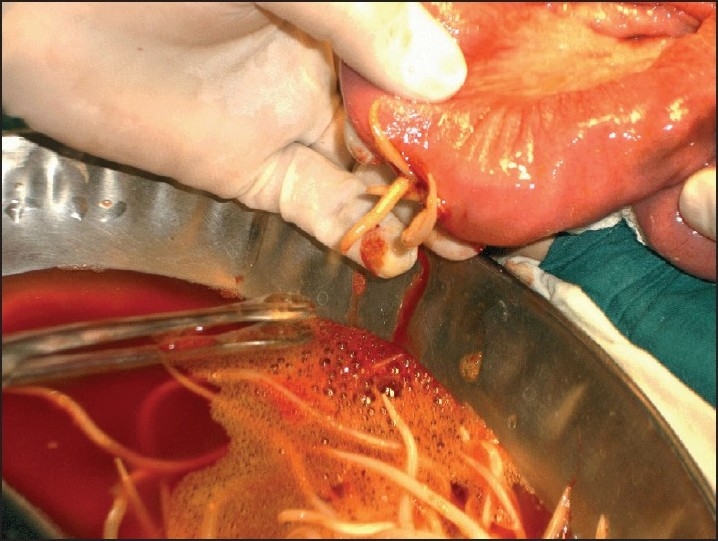
Enterotomy being done for impacted worm bolus in a child.

**Figure 3:**
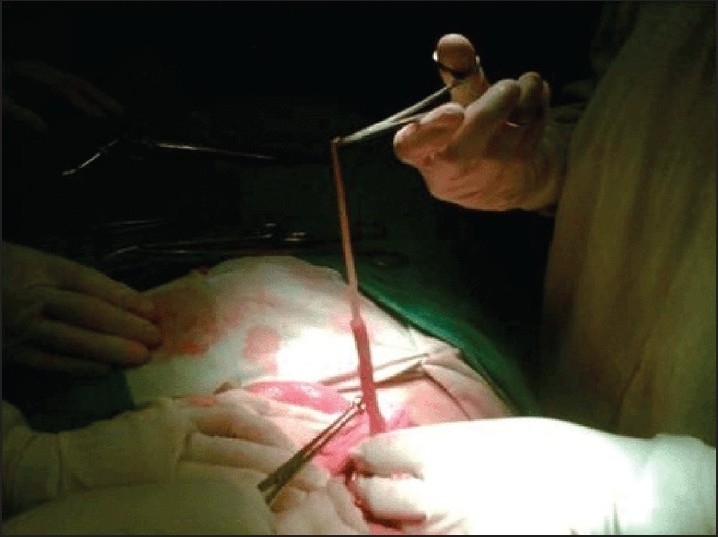
*Ascaris lumbricoides* being removed through tip of vermiform appendix in grossly normal appendix which has no evidence of any appendicitis at histopathology

**Figure 4:**
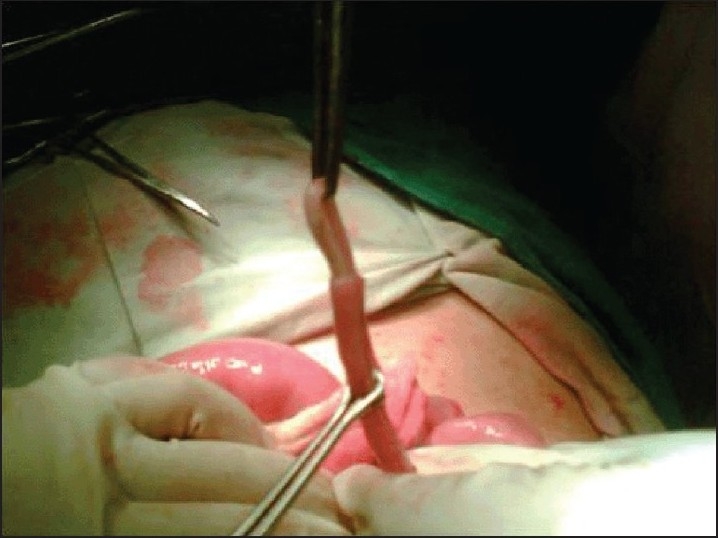
Second worm being removed through above normal appendix.Worm lying with tail end at tip of appendix, held at tail end and being removed.

**Figure 5:**
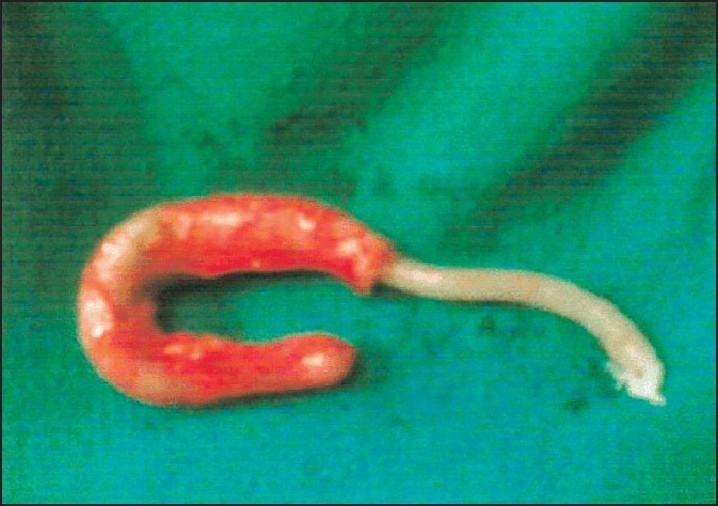
*Ascaris lumbricoides* with head end at base of appendix in a grossly inflammed appendix which had features of ascaridial appendicitis on histopathology.

**Figure 6:**
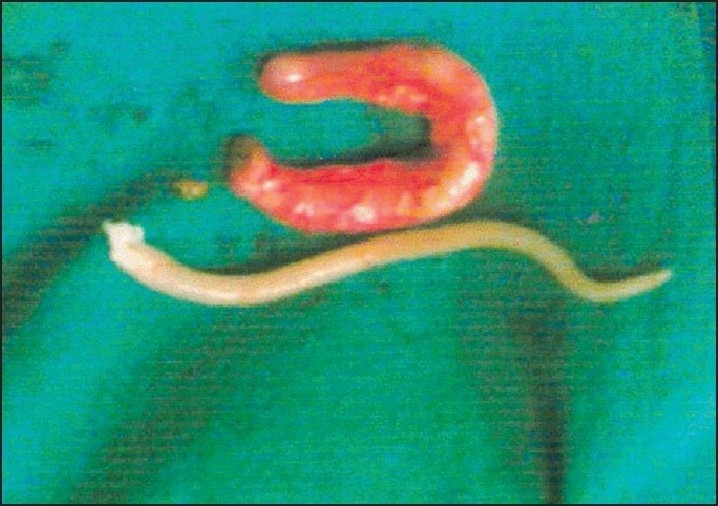
*Ascaris lumbrocoides* which was lying with tail end lying at the tip and head end at base of grossly inflammed vermiform appendix.

**Figure 7:**
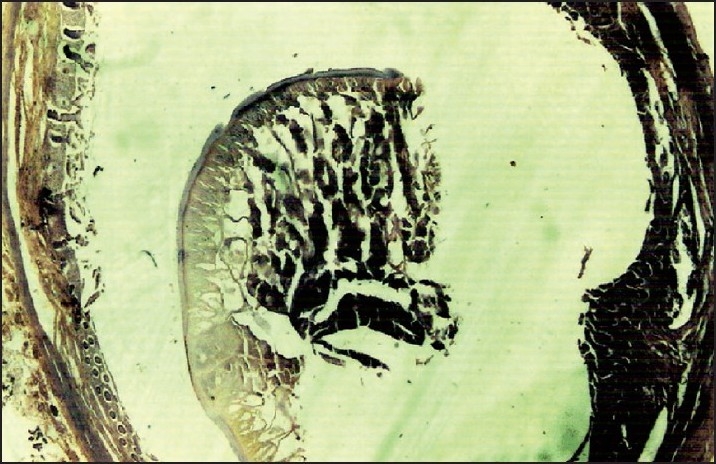
Cross section of vermiform appendix having *Ascaris lumbrocoides* in lumen; no features of appendicitis can be seen.

Worms were found incidentally in the appendix of five patients (45.4%) who had kneading of worms, in two patients (18%) who had enterotomy for worms (Figures 1–4), during appendectomy for appendicitis for two patients (18%) (Figures 5 and 6), during the course of incidental appendectomy in one patient (9%) who had adhesional intestinal obstruction, and in one patient (9%) who had appendectomy for perforated appendix. The number of worms in the appendix ranged from one to three worms. A single worm was found in the vermiform appendix in nine patients (81.8%) and three worms were found in the vermiform appendix in one patient (9%). A characteristic finding was that worms in the appendix had their heads at the base and their tails at the tip of the appendix. One patient (9%) had five worms in the peritoneal cavity after perforation of the appendix. Only three patients (27.2%) had histopathologically documented evidence of *Ascaris*-associated appendicitis while the other eight cases were found to have normal appendix on histopathological examination (Figure 7). Laparotomy revealed worms in the appendix of three patients (27.2%) through the appendiceal wall. Live worms were observed whenever a diagnosis was made intraoperatively.

## DISCUSSION

Surgical manifestations of abdominal ascariasis are varied and are attributed to the wandering nature of *Ascaris lumbricoides*. The preoperative diagnosis of this condition continues to remain difficult, although the parasite can sometimes be observed inside the lumen during micropathological examination. Appendicitis due to the migration of *Ascaris lumbrocoides* into the appendix is still debatable because the symptoms of this migration may simulate appendicitis, but rarely cause it.^3^,^4^ The hypothesis that *Ascaris* lumbricoides is a major cause of appendicitis in children has been disproved.^5^

In *Ascaris* infestations associated with a normal appendix, *Ascaris* lodges in the appendix and comes and goes on its own accounting for the intermittent pain observed sometimes in children with high worm load. During the kneading of the worms, this high intestinal worm load coupled with a competent illeaocecal valve can sometimes provide a high load of worms in the cecum. This leads to the entry of the worms into the lumen of the appendix to escape the kneading. A competent ileocecal valve prevents the worms from escaping through the retrograde route. An incompetent ileocecal valve with proximal worm bolus obstruction may force the worm to travel again towards the cecum. This further contributes to the worm load in the cecum and in an attempt to seek natural orifices, the worms may enter the vermiform appendix. This type of *Ascaris*-associated normal appendix occurs in the wide-lumen, free-lying appendix. More than one worm can be seen in the lumen even when there are no grossly or microscopically visible features of appendicitis. An inflamed appendix can contain worms inside its lumen although it is debatable whether the worms caused the inflammation or whether they migrated to an already inflamed appendix. However, the presence of live worms and the associated pathology of the appendix do not favor the hypothesis that the worms cause appendicitis. Also, the presence of *Ascaris* inside the inflamed appendix favors the hypothesis that *Ascaris* has an affinity for pathological tissue. The wandering nature of *Ascaris lumbricodes* makes these worms seek openings just as they do in the perforated appendix wherein they reach the perforation site and lodge freely in the peritoneal cavity.

One of the characteristic findings of this study was that the worms were seen in the appendix with their heads at the base and their tail ends at the tip end of the appendix, which might lead to the frequent escape of worms from the appendix. *Ascaris* can be removed through the distal tip of the appendix when more than one worm is seen inside the appendix. It is to be stressed that complete removal of worms from the appendix is to be done when only a portion of the worm is lying inside the appendix and part of it is inside the cecum to avoid necrosis of the portion inside the appendicular stump, which may lead to fecal fistula. Our observations support the direct evidence of the presence of *Ascaris* in the vermiform appendix in contrast to reports of indirect evidence of migration of the worms into the appendix due to the presence of *Ascaris lumbricoides* eggs lodged in the appendix without any features of appendicitis.^6^

In conclusion, *Ascaris lumbrocoides* is rarely found in the appendix and its presence there is rarely associated with appendicitis. Worms in the appendix can be extracted through the distal tip of the appendix. *Ascaris* position themselves with their heads directed towards the base and their tail ends at the tip of the vermiform appendix.
